# Supplementary material for: Silver Nanoparticle-Intercalated Cotton Fiber for Catalytic Degradation of Aqueous Organic Dyes for Water Pollution Mitigation
Source: Nanomaterials (Basel). 2022 May 10;12(10):1621. doi: 10.3390/nano12101621 (PMC9142960; doi:10.3390/nano12101621)
Supplement: Supplementary file 1 [file nanomaterials-12-01621-s001.zip › nanomaterials-1680974-supplementary.pdf]

nanomaterials

Supplemental Material

Silver Nanoparticle-Intercalated Cotton Fiber for Catalytic Degradation of Aqueous Organic Dyes for Water Pollution Mitigation

Matthew Blake Hillyer, Jacobs H. Jordan, Sunghyun Nam *, Michael W. Easson and Brian D. Condon

Cotton Chemistry and Utilization Research Unit, Southern Regional Research Center, Agricultural Research Service, United States Department of Agriculture, New Orleans, LA 70113, USA; matthew.hillyer@usda.gov (M.B.H.); jacobs.jordan@usda.gov (J.H.J.); michael.easson@usda.gov (M.W.E.); brian.condon@usda.gov (B.D.C.)

* Correspondence: sunghyun.nam@usda.gov; Tel.: +1-(504)-286-4229

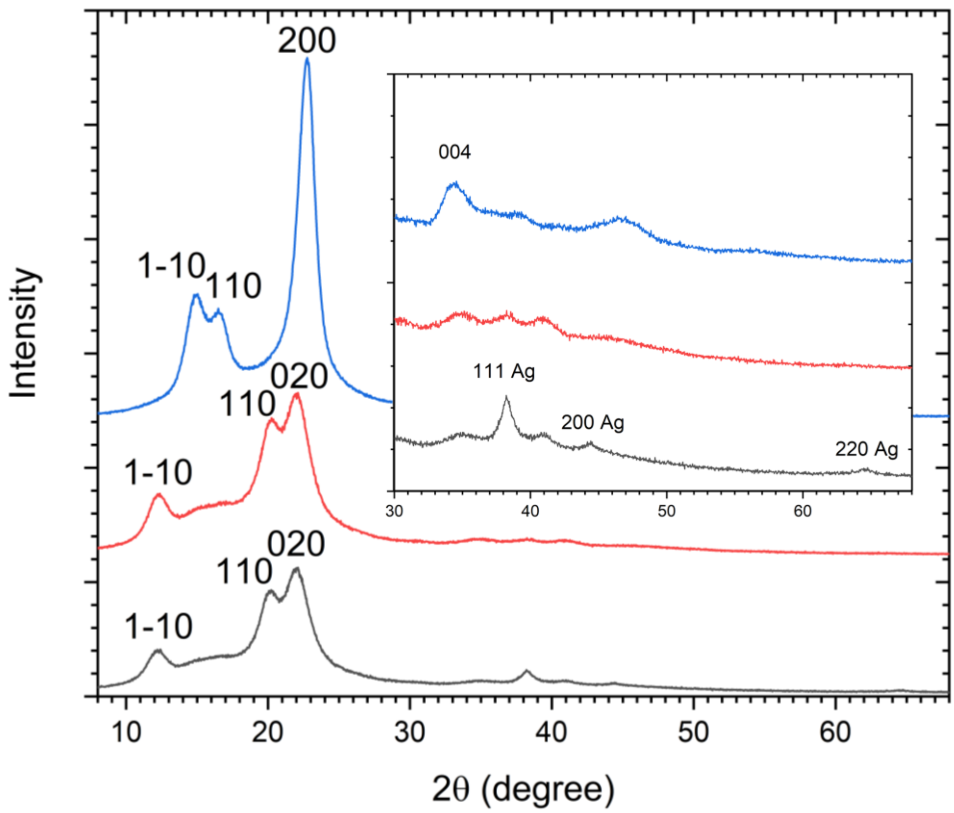

Figure S1. XRD spectra of (blue) pristine white cotton, (red) mercerized white cotton, and (black) Ag NP cotton catalyst with (inset) focused view of the spectra between 30 and 68°. Peaks for the cellulose from cotton and Ag NP lattice planes are denoted.

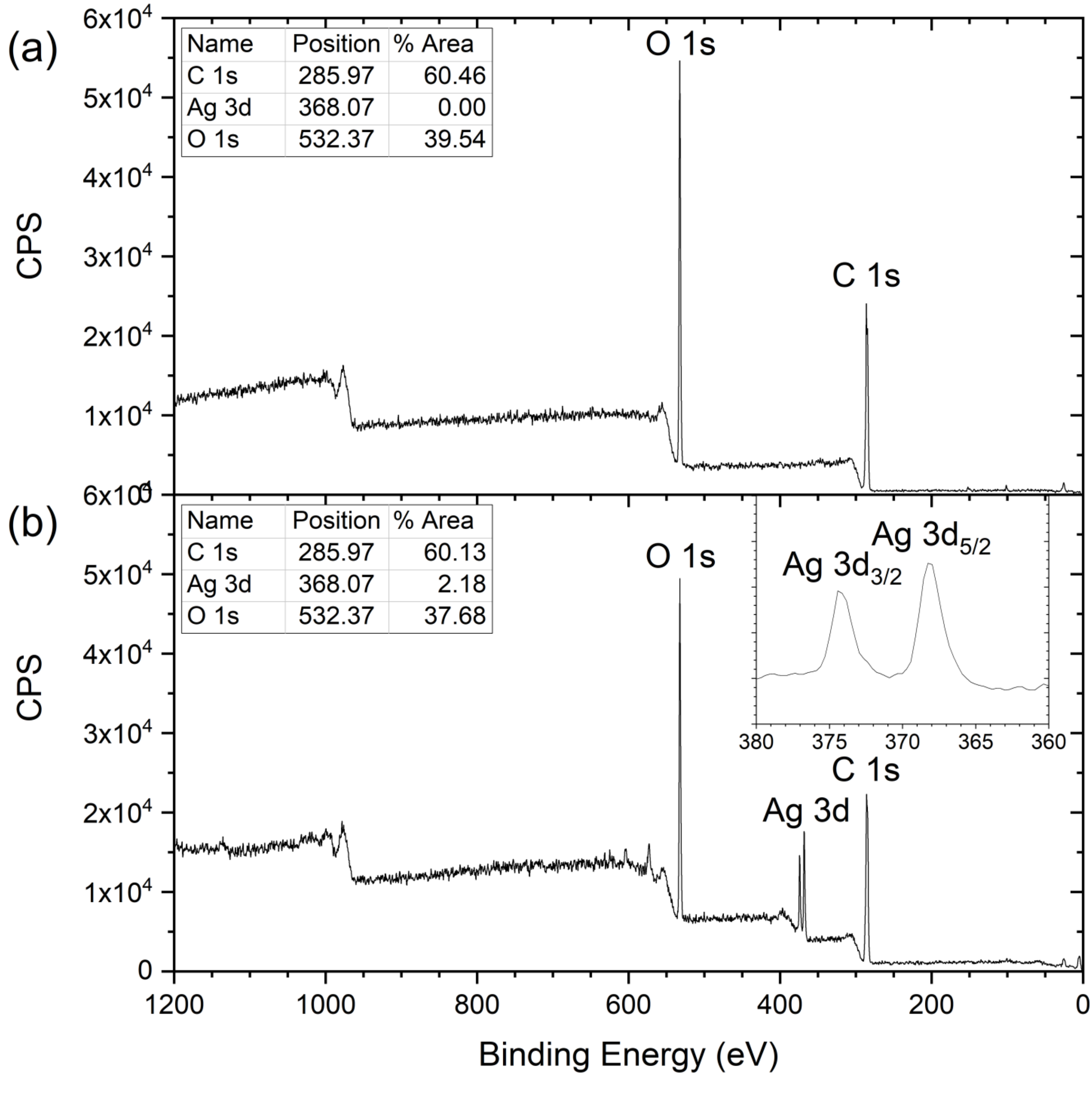

Figure S2. XPS spectra of (**a**) pristine white cotton and (**b**) Ag NP cotton catalyst with inset of Ag $3d_{3/2}$ and Ag $3d_{5/2}$.

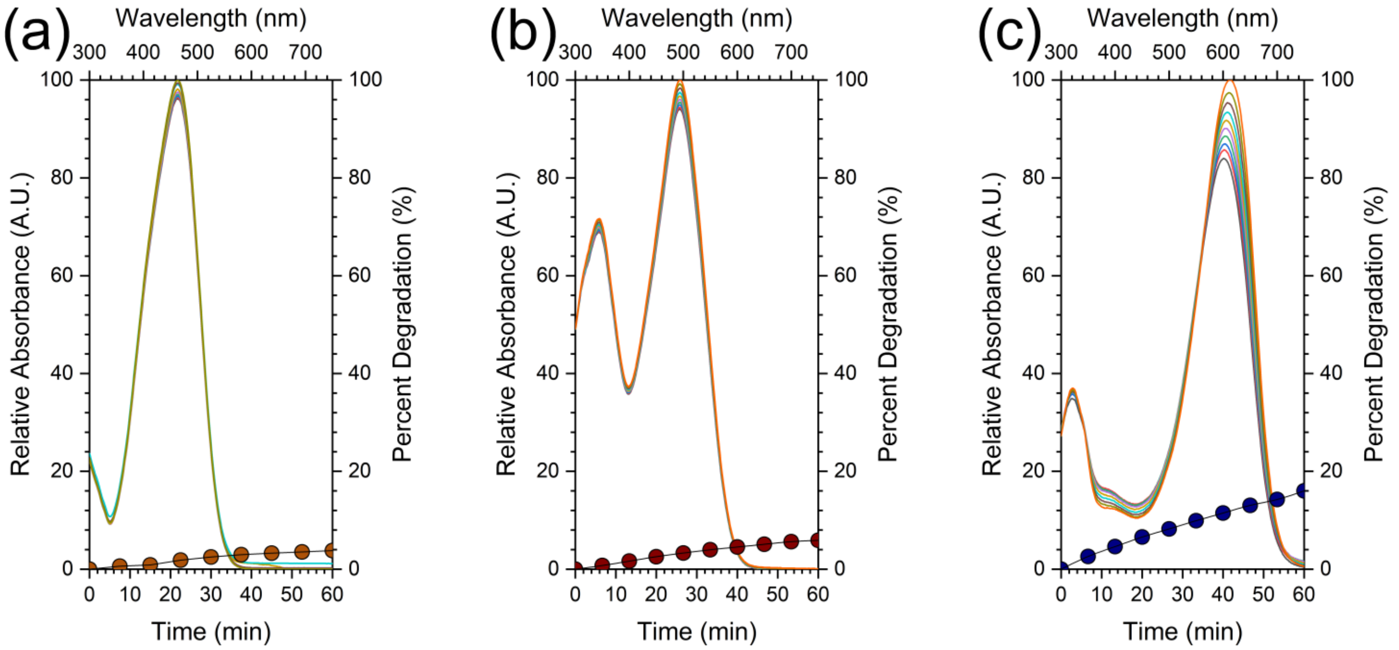

Figure S3. UV/vis spectra and overlay of percent degradation of dyes (25 ppm) excess $NaBH_4$ in the absence of Ag NP-cotton catalyst at pH 7 for (**a**) MO, (**b**) CR, and (**c**) CSBB.

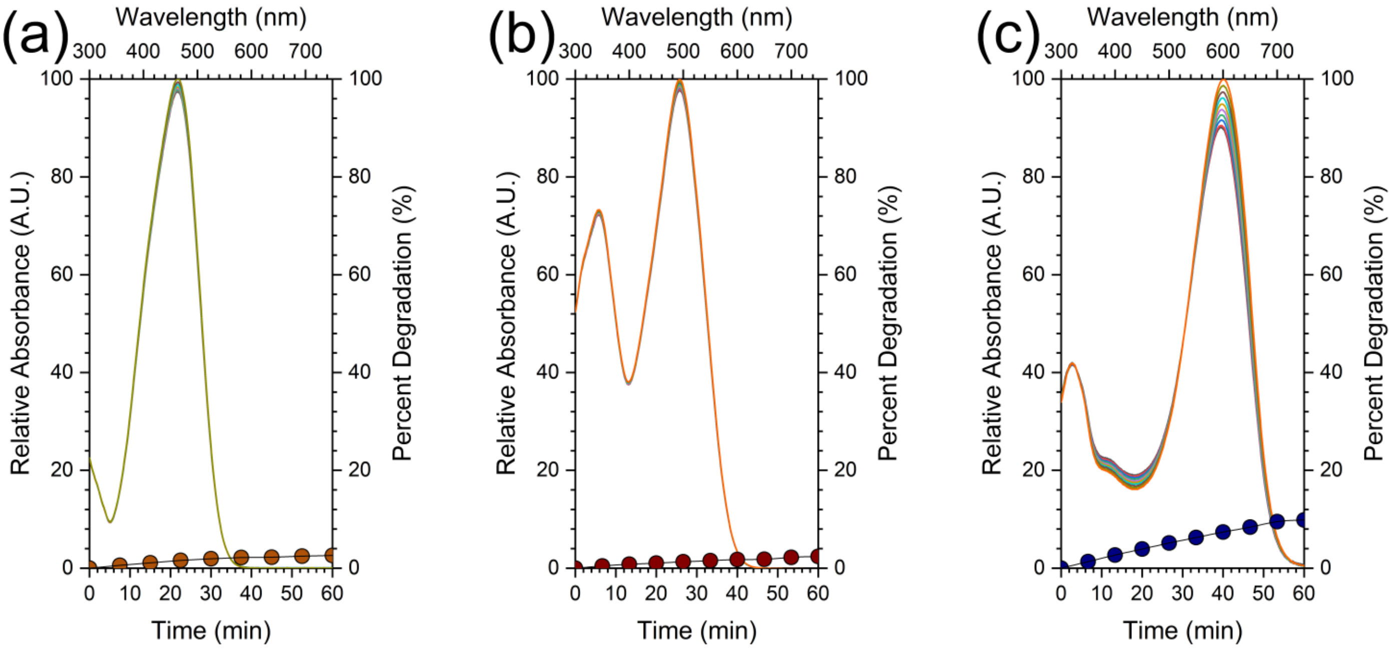

Figure S4. UV/vis spectra and overlay of percent degradation of dyes (25 ppm) excess $NaBH_4$ in the absence of Ag NP-cotton catalyst at pH 9 for (**a**) MO, (**b**) CR, and (**c**) CSBB.

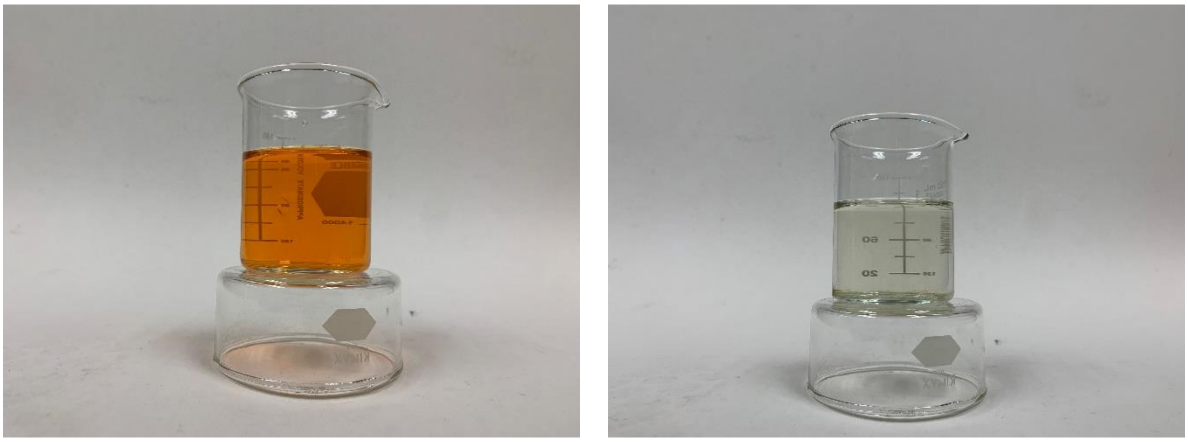

Figure S5. Digital images degradation reactions for MO at 25 ppm (left) before reaction and (right) after reaction time.

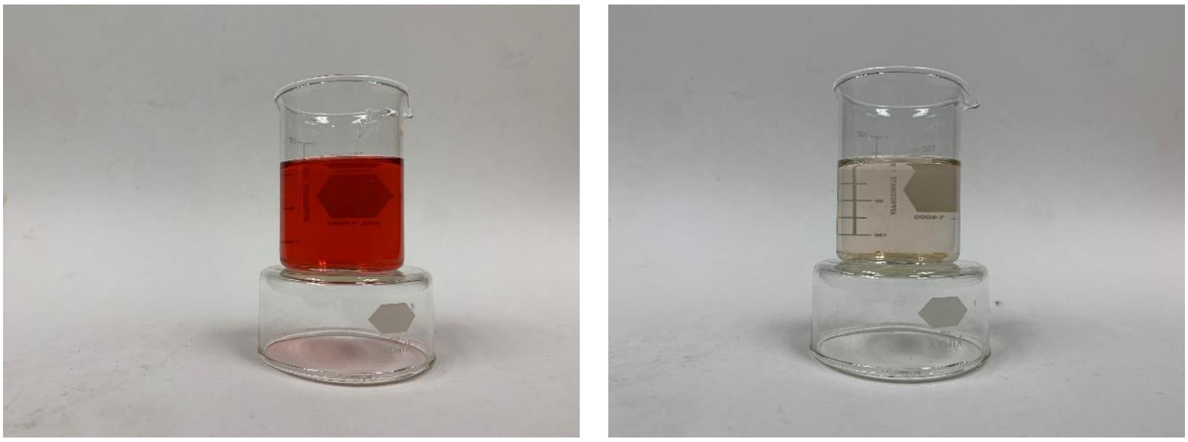

Figure S6. Digital images degradation reactions for CR at 25 ppm (left) before reaction and (right) after reaction time.

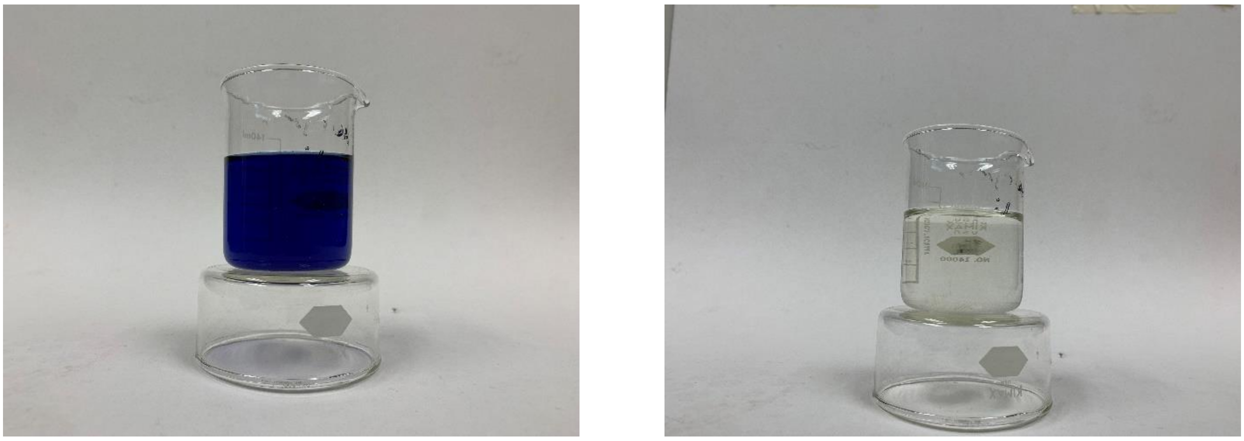

Figure S7. Digital images degradation reactions for CSBB at 25 ppm (left) before reaction and (right) after reaction time.

Table S1. Comparison of degradation reaction conditions for this study and others similarly performed in the literature.

[Ag NP] (µg/mL)	Dye	[Dye] (ppm)	[NaBH4] (mM)	Time (min)	Reference
26.3	Methyl Orange	25	7.93	90	This study
26.3	Congo Red	25	7.93	145	
26.3	Chicago Sky Blue 6B	25	7.93	125	
52.6	Methyl Orange	25	7.93	30	
52.6	Congo Red	25	7.93	18.5	
52.6	Chicago Sky Blue 6B	25	7.93	21	
100	Methyl Orange	33	100	3	[1]
1	Methyl Orange	16	135 *	40	[2]
0.108	Congo Red	7	1	50	[3]
4	Congo Red	581	83	15	[4]

* Peroxodisulfate as reductant.

References

1. Bogireddy, N.K.R.; Kumar, H.A.K.; Mandal, B.K. Biofabricated silver nanoparticles as green catalyst in the degradation of different textile dyes. *J. Environ. Chem. Eng.* **2016**, *4*, 56–64, https://doi.org/10.1016/j.jece.2015.11.004.
2. Nagar, N.; Devra, V. A kinetic study on the degradation and biodegradability of silver nanoparticles catalyzed Methyl Orange and textile effluents. *Heliyon* **2019**, *5*, e01356, https://doi.org/10.1016/j.heliyon.2019.e01356.
3. Albeladi, S.S.R.; Malik, M.A.; Al-Thabaiti, S.A. Facile biofabrication of silver nanoparticles using Salvia officinalis leaf extract and its catalytic activity towards Congo red dye degradation. *J. Mater. Res. Technol.* **2020**, *9*, 10031–10044, https://doi.org/10.1016/j.jmrt.2020.06.074.
4. Kolya, H.; Maiti, P.; Pandey, A.; Tripathy, T. Green synthesis of silver nanoparticles with antimicrobial and azo dye (Congo red) degradation properties using Amaranthus gangeticus Linn leaf extract. *J. Anal. Sci. Technol.* **2015**, *6*, 33, https://doi.org/10.1186/s40543-015-0074-1.
